# Discovery of miRNAs and Their Corresponding miRNA Genes in Atlantic Cod (*Gadus morhua*): Use of Stable miRNAs as Reference Genes Reveals Subgroups of miRNAs That Are Highly Expressed in Particular Organs

**DOI:** 10.1371/journal.pone.0153324

**Published:** 2016-04-29

**Authors:** Rune Andreassen, Fredrik Rangnes, Maria Sivertsen, Michelle Chiang, Michelle Tran, Merete Molton Worren

**Affiliations:** 1 Department of Pharmacy and Biomedical Laboratory Sciences, Faculty of Health Sciences, Oslo and Akershus University College of Applied Sciences, Oslo, Norway; 2 Bioinformatics Core Facility, Institute for Medical Informatics, Oslo University Hospital, Oslo, Norway; Institut National de la Recherche Agronomique (INRA), FRANCE

## Abstract

**Background:**

Atlantic cod (*Gadus morhua*) is among the economically most important species in the northern Atlantic Ocean and a model species for studying development of the immune system in vertebrates. MicroRNAs (miRNAs) are an abundant class of small RNA molecules that regulate fundamental biological processes at the post-transcriptional level. Detailed knowledge about a species miRNA repertoire is necessary to study how the miRNA transcriptome modulate gene expression. We have therefore discovered and characterized mature miRNAs and their corresponding miRNA genes in Atlantic cod. We have also performed a validation study to identify suitable reference genes for RT-qPCR analysis of miRNA expression in Atlantic cod. Finally, we utilized the newly characterized miRNA repertoire and the dedicated RT-qPCR method to reveal miRNAs that are highly expressed in certain organs.

**Results:**

The discovery analysis revealed 490 mature miRNAs (401 unique sequences) along with precursor sequences and genomic location of the miRNA genes. Twenty six of these were novel miRNA genes. Validation studies ranked *gmo*-miR-17-1—5p or the two-gene combination *gmo*-miR25-3p and *gmo*-miR210-5p as most suitable qPCR reference genes. Analysis by RT-qPCR revealed 45 miRNAs with significantly higher expression in tissues from one or a few organs. Comparisons to other vertebrates indicate that some of these miRNAs may regulate processes like growth, lipid metabolism, immune response to microbial infections and scar damage repair. Three teleost-specific and three novel Atlantic cod miRNAs were among the differentially expressed miRNAs.

**Conclusions:**

The number of known mature miRNAs was considerably increased by our identification of miRNAs and miRNA genes in Atlantic cod. This will benefit further functional studies of miRNA expression using deep sequencing methods. The validation study showed that stable miRNAs are suitable reference genes for RT-qPCR analysis of miRNA expression. Applying RT-qPCR we have identified several miRNAs likely to have important regulatory functions in particular organs.

## Introduction

Atlantic cod (*Gadus morhua*) is an economically important marine fish species for wild fisheries of the northern Atlantic with more than 1 million tonnes captured in commercial fisheries in 2014 [1]. Although there are challenges, the Atlantic cod is also considered an interesting species to the aquaculture industry. The commercial importance of Atlantic cod has led to the development of several genomic resources to better understand cod biology and how genetic variation may affect economically important traits [2–6]. Recently, a sequencing project using next generation sequencing and a whole genome shotgun approach was completed. The genome sequence assembled from these data represents a first Atlantic cod reference genome sequence publicly available and allows for miRNA discovery by use of deep sequencing methods [7].

MicroRNAs (miRNAs) are an abundant class of endogenous small RNA molecules that downregulate gene expression at the post-transcriptional level [8–10]. Most miRNAs are highly conserved among vertebrates with limited sequence variation across species. There are, on the other hand, also non-conserved miRNAs that are present in one species only (species specific miRNAs) [11, 12]. Evolutionary conserved miRNA genes are not only similar in their sequences among species. Some miRNA genes are closely located in the genome (clustered), and such miRNA gene clusters are also often conserved across species. Furthermore, if a miRNA gene cluster is located in an intron the clustered miRNA genes are often co-expressed with the host-gene [13, 14].

A prerequisite to study miRNAs and their functions in new species would be to discover and characterize the sequences of their biologically active mature miRNAs as well as the sequence and location of their corresponding miRNA genes. Recent advances in sequencing technology have led to increased sensitivity in sequencing analysis (deep sequencing) that allows even lowly abundant small RNAs to be detected [15]. Applying bioinformatics tools that utilize deep sequencing data together with current knowledge of the biosynthesis of miRNAs and a reference genome, both conserved miRNAs as well as novel miRNAs, may be discovered in a new species [15, 16]. Such miRNA discovery and characterization studies have been carried out in a large number of commercially important teleost species over the recent years (e.g. [17–20]). Recently the first miRNA discovery study was performed in Atlantic cod with a particular focus on characterization of miRNAs present in developmental tissues and their expression differences associated with temperature [21].

MiRNAs are major regulators of protein coding gene expression in vertebrates, and they act as key regulators of fundamental biological processes like growth, immune response, tissue development and maintenance of tissue specific functions [22–26]. They may also act as causative mediators of disease. This has led to studies of how miRNAs affect health in humans as well as in other common model organisms [27, 28]. The motivation for miRNA research in commercially important teleost species would, however, not only be restricted to studies on how they affect health and disease, but would also include studies on how miRNAs may affect economically interesting traits like growth or food conversion. To fully understand the interaction between genes in networks that affect such interesting traits it is necessary to identify the miRNAs that participate as key regulators [29–32]. The miRNAs that show organ specific expression patterns are often key regulators of biological functions carried out by the organ in which they are uniquely expressed [13, 33]. Thus, to disclose which miRNAs that are particularly important in development and function of various tissues a first step could be to identify the miRNAs that are highly expressed in one or few particular organs. There have been relatively few studies aiming at identifying such miRNAs in non-model teleosts [34].

The aims of this study is to discover and characterize miRNAs in Atlantic cod as well as reveal those miRNAs that are expressed at significantly higher levels in particular organs. Most known mature miRNAs identified so far were discovered in tissues from early developmental stages [21]. Our discovery analysis was, therefore, carried out independently in eight organ samples from a 1 kg individual to enable identification of a large number of miRNAs with a high confidence. To facilitate analysis of differentially expressed miRNAs in Atlantic cod by RT-qPCR we carried out a validation study to identify miRNAs suitable as endogenous controls in this species. Finally, the expression profiles obtained from the deep sequencing datasets and the validated RT-qPCR method were utilized to identify a subset of miRNAs with higher expression in certain organs.

## Material and Methods

### Small RNA isolation from eight individuals

One wild-caught coastal Atlantic cod individual was used for deep sequencing, whereas samples from additional seven wild-caught coastal Atlantic cod individuals (a total of eight individuals) were used for qPCR. The individuals (0.6–1 kg) were from the Oslofjord, south-east Norway. No specific permissions were required for sampling fish at these locations and the field studies did not involve endangered or protected species. The fish were provided by local fishermen. The one individual that was used for small RNA deep sequencing (a female) was caught, kept alive in a saltwater tank, sacrificed in the harbour and immediately given to us. The remaining seven individuals (three males) were caught, sacrificed immediately upon capture and put on ice. Thus, the one individual that was used for deep sequencing (see next section of Material and Methods) was kept on ice less than 30 min before dissected, the other seven individuals were kept on ice less than 3 hours before dissected. Dissection of fish and sampling of materials was performed in agreement with the provisions enforced by the Norwegian Animal Research Authority. As fish were sacrificed prior to our study and delivered by local fishermen no permission was required from the Institutional Animal Care and Use Committee (IACUC). Immediately after dissection samples were collected from each of the individuals to RNA later (Ambion, Life technologies, Carlsbad, CA, USA) for isolation of total RNA. The samples were from nine different organs: head-kidney, heart, brain, gills, skeletal muscle, liver, spleen, skin and intestine. Thus, each sample consisted of tissues from a particular organ. Samples of all nine organs were taken from all eight individuals dissected. A sample of about 0.05 g was collected from each organ and stored in RNA later. The sample from the intestine was flushed with distilled water to remove exogenous material before stored in RNA later. The skin samples were rectangular pieces sampled close to the lateral line. Any muscle tissues were removed from the skin samples before they were stored in RNA later.

Total RNA was isolated from each of the samples by use of the mirVana miRNA isolation kit (Ambion, Life technologies, Carlsbad, CA, USA) following the manufacturer’s protocol. The RNA concentration and purity were determined using the Bioanalyzer and/or Nanodrop following the manufacturer’s protocol. The 72 samples (eight individuals, nine tissues) showed concentrations of total RNA ranging from 35–900 ng/μl (total volume 100 μl). All 72 samples were used for RT-qPCR analysis of miRNA expression.

### Pre-processing, deep sequencing and miRDeep analysis of deep sequencing data

Samples from one of the eight individuals (see above) were selected for deep sequencing and tissue from the following organs were included: head-kidney, brain, gills, skeletal muscle, liver, spleen, skin and intestine. The concentration of total RNA in these particular samples ranged from 75–865 ng/μl (total volume 100 μl) and with RIN values ≥ 7, see S1 Table. The Illumina TruSeq Small RNA Library Preparation Kit (Illumina, San Diego, USA) was used in the preparation of the libraries as described by the manufacturer with 1 ug total RNA input. After adapter ligation and cDNA synthesis the products were purified on a gel and the fraction between 145–160 bp was used for sequencing. The library construction was performed at the Norwegian Genomics Consortium’s genomics core facility. The small RNA libraries constructed from the eight tissues were successfully subjected to next generation sequencing using Illumina Genome Analyzer IIx sequencing platform as described in Andreassen et al.[19]. The sequence reads (fastq-files) from each of the eight samples showed reads with Q-values ≥32. Adapter only sequences (5’TGGAATTCTCGGGTGCCAAGGAACTCCAGTCAC 3’) were removed, reads were trimmed (removal of nucleotides from adapter sequences) and reads were size filtered to remove reads less than 18 nucleotides by use of the fastx-clipper tool (http://hannonlab.cshl.edu/fastx_toolkit/). The miRNA discovery analysis was independently performed in each of the eight samples to allow for detection of miRNAs highly expressed in particular tissues. The genome assembly from the *Gadus morhua* whole genome shotgun sequencing project (http://hgdownload.soe.ucsc.edu/goldenPath/gadMor1/bigZips/), GenBank accession number: CAEA00000000.1, was used as reference genome. The high quality, adapter processed reads were used as the experimental data, and the discovery analysis was performed using the miRDeep2 software package (mapper and miRDeep2 analysis modules) [15, 35]. Default commands were used in the miRDeep2 analysis except that conservation scoring was omitted and the parameter g was set to -1 to allow all precursors to be analyzed during automatic excision gearing. We used the miRDeep2 score that yielded a signal-to-noise ratio of 30:1 as a cut-off threshold. All precursors with scores above this threshold and with reads that aligned perfectly, and in a discrete manner, to both 5’ and 3’ end of a precursor were regarded as putative miRNAs. These putative precursor sequences were further analyzed by BLAST searches against all known precursor sequences deposited in miRBase, release 21 (http://www.mirbase.org/search.shtml). We defined a significant hit as a match with an E-value ≤ 1E-06 to a stem-loop in miRBase. Any putative miRNA precursor sequence that provided a significant hit in the BLAST analyses was accepted as a true *Gadus morhua* miRNA precursor sequence, and each of these were annotated as the evolutionary conserved *Gadus morhua* ortholog of the miRNA gene in miRBase that retrieved the best hit. There are, at present, no miRNAs from *Gadus morhua* in the current version of miRBase, but Atlantic cod miRNAs have recently been characterized in materials from developmental stages [21] and the results submitted to miRBase. To facilitate comparison between our study and Bizuayehu et al [21] and to ensure that annotation are in agreement with the nomenclature guidelines [11, 36], our results from discovery and characterization of miRNAs were submitted to miRBase. The final annotation of all miRNAs and miRNA precursor sequences given in the results section was carried out by miRBase.

The precursors that were identified by miRDeep, but did not significantly match any miRNA precursor in miRBase were considered as putative novel miRNAs. All such precursors were used as queries in BLAST analysis that were performed against the nt/nr and refseqRNA databases in Genbank (http://blast.ncbi.nlm.nih.gov/Blast) and the small RNA family database in Rfam (http://rfam.xfam.org/search). Any putative precursor that showed a significant hit against these databases were considered to be other kinds of small RNA and excluded. Finally, all precursors were used as queries in BLAST analysis against the *Gadus morhua* genome sequence (http://www.ensembl.org/Gadus_morhua/Tools/Blast?db=core). Any putative precursor with a significant BLAST hit, defined as E-value ≤ 1E-06 against multiple loci (>5) in the genome reference sequence were considered to be part of interspersed repeats or tandem repeats and, consequently, excluded as novel miRNAs. The remaining putative novel miRNAs were validated in the following manner: they should be detected in at least two independent deep sequencing samples. More than five reads from the samples sequenced should match perfectly the expected mature products from both arms (5p and 3p), and the reads that aligned to the precursor should support that there was a consistent processing of the 5’end of the mature sequences. Passing all these criteria they were considered to be true novel *Gadus morhua* miRNAs.

The presence of clustered miRNA genes among the miRNA genes discovered in our study was investigated by comparing precursor locations within contigs. Any two miRNA precursors located within the same contig, less than 10 kb apart and with same direction of the transcription was considered part of a miRNA gene cluster. This definition (10 kb) is the same as the one used as default in miRBase (http://www.mirbase.org/search.shtml).

Sequencher software 5.3 (Gene Codes Corporation, Ann Arbor, USA) was used to align mature miRNA sequences (5p or 3p). By applying strict settings only identical mature sequences were allowed to align, thus, providing the total number of unique mature miRNAs in our materials.

### cDNA synthesis and RT-qPCR

The miScript assays were used for cDNA synthesis and qPCR as described by the manufacturer (Qiagen, Hilden, Germany). A universal primer (reverse primer), provided with the miScript qPCR kit, was used in combination with our own custom designed forward primer in the qPCR assays amplifying each of the mature miRNAs. The sequence of the mature miRNAs characterized in this study was utilized to produce miRNA-specific forward primers. All primers were purchased from Sigma Aldridge, purified by desalt only and provided as liquid solution of 100 μM from the manufacturer. They were diluted to 10 μM for use in each of the qPCR assays. All forward primer sequences are given in S2 Table. The qPCR analysis was run on an Mx3000p (Stratagene). The qPCR reaction mixture consisted of 12.5 μL 2xQuantitec Syber Green Master Mix, 2.5 μL 10x miScript Universal Primer, 2.5 μL of 10 μM forward miRNA-specific primer, 5 μL Rnase free water, and 2.5 μL cDNA (template). The following program was used in qPCR: one thermal cycle at 95°C for 15 min followed by 40 cycles of 94°C for 15 sec, 55°C for 30 sec and 70°C for 30 sec. The Mx3000p software package was used for qPCR analysis. The cybergreen assay module includes a final melting point analysis that follows the 40 cycles of quantitative PCR. Plots from melting point analysis were manually inspected for all the miRNA assays tested to verify that forward primers were specific (data not shown).

### Normalised read counts from deep sequencing datasets

The deep sequencing data from eight samples (S1 Table) was used to provide crude estimates of expression of individual miRNAs across different tissues. First, the mature miRNA sequences discovered and annotated in this study (from miRDeep) were used to generate an index file for NovoAlign software (http://www.novocraft.com). Novoalign was then used to count the number of miRNA reads with a perfect match to any mature miRNA in each of the trimmed and size filtered deep sequencing samples. To obtain normalized read counts the total counts of a particular miRNA (reads in sizes >18 nt) was divided by the total number of all miRNAs in that sample and multiplied with 10^4^. These normalized read counts of individual miRNAs could be compared across samples and were, thus, utilized to select candidate miRNAs that were likely to have higher expression in certain organs or candidate miRNAs that showed very similar expression level across all tissues (candidate reference genes). The read counts and normalized read counts from Novoalign analysis is given in S3 Table.

### Stability testing of candidate reference genes

The average normalised read counts of each of the mature miRNAs across the eight samples and their standard deviation was estimated and utilized for comparison of individual miRNA stability across tissues. The relative standard deviation (RSD, coefficient of variation) was estimated by dividing the standard deviation of mean normalised read count from each mature miRNA by their mean. The RSD of each mature miRNA was used as a measurement of their relative stability across the tissues. Avoiding those miRNAs that were part of miRNA gene families (close sequence similarity) or belonging to same gene clusters [37] a set of candidate genes with low RSDs were selected for further stability testing by RT-qPCR. The commonly used endogenous control snRNA U6 was also tested in seven random samples (two brain, two intestine, head kidney, liver, heart and spleen samples) using the commercially available miScript assay MS00033740 (Qiagen, Hilden, Germany). The efficiency of the U6 assay was 57% and the Ct values showed rather low concentrations of U6 (n = 7, average Ct = 28,3). This indicated that U6 was not suitable as reference gene, and, consequently, it was not included as a candidate reference gene in NormFinder analysis (see below). The stability (standard deviation) from measurements of the seven samples using U6 was, however, compared to the stability of the best performing miRNAs.

The candidate reference miRNAs were analyzed by RT-qPCR in all samples from the nine different tissues (n = 72). All raw Ct values were transformed to linear scale and grouped by tissues before used as input in NormFinder. The NormFinder algorithm (http://moma.dk/normfinder-software) [38] calculates a “stability value” that is inversely correlated with the stability of gene expression (so a higher stability value indicates lower stability). This data normalization tool was used to rank the expressions of candidate genes by their stability and to identify the most suitable reference genes for normalization of RT-qPCR measurements of miRNA expression in Atlantic cod.

### Identification of differentially expressed miRNAs by RT-qPCR

The normalized read counts of individual miRNAs from the deep sequencing samples was utilized to select miRNAs that were likely to have higher expression in one or more of the tissue samples. A total of 47 miRNAs were selected for RT-qPCR analysis from this manual comparison. In addition, a few evolutionary conserved miRNAs known to exhibit tissue enriched expression in other vertebrates (*gmo*-miR-736, *gmo*-miR-499 and *gmo*-miR-26a) were also selected for qPCR analysis. Thus, a total of 50 miRNAs were selected for RT-qPCR. All eight samples from the tissue(s) where a given miRNA was suspected to be expressed at higher levels (target tissue) were analysed together with four samples from each of the other tissues (reference tissues) in the same qPCR run. The Ct-value of a putative differentially expressed miRNA was normalized by subtracting the geometric mean of Ct-values generated from reference genes. The normalized measurements (ΔCts) were compared for all the tissues tested, an approach similar to Liang et al. [13]. The four normalised Ct-values (ΔCt) from each different tissue in the reference group were inspected to make sure they showed low and non-overlapping expression levels compared to target tissue(s). This inspection revealed that in some cases the selected miRNAs seemed to have higher expression in more tissues than the initially selected target tissue. If so, these additional tissues were also defined as target tissues. The number of samples analysed in the reference group, thus, was from 20 to 32 samples depending on how many tissues that were defined as target and reference tissues for a given miRNA. The mean normalized Ct and the spread (standard deviation) was calculated for each of the groups, and Welchs t-test was used to test whether there was a significant difference in expression in target tissue vs reference tissue of a given target miRNA. A total of 76 tests were performed and the significant levels were adjusted according to number of tests to a “Bonferroni corrected” p-value of 6,6E-04. The relative increase in expression in target tissue of each miRNA was calculated using the principle of the comparative Ct method (ΔΔCt- method) [39]. Applying this method the average relative increase in target tissue could be calculated as the difference between mean normalized Ct from target tissue and mean normalized Ct from reference tissue. A correlation analysis between relative increases estimated from deep sequencing data and from RT-qPCR was performed. Those miRNAs that could be quantified in all eight deep sequencing samples (normalized read numbers larger than zero, a total of 34) were included in this comparison.

## Results

### Discovery of miRNAs and miRNA genes by deep sequencing and miRDeep analysis

The number of quality filtered (quality score above 32) and adapter trimmed reads from each sample ranged from 1.8 to 6.5 million. The concentration of total RNA and RIN values of the eight samples submitted for deep sequencing is given in S1 Table. This file also gives an overview of results from deep sequencing with total number of reads, number of adapter only reads, the total number of quality filtered, adapter trimmed and size filtered reads and total number of unique reads for each of the samples. All data from deep sequencing of the eight samples have been submitted to the NCBI Sequence Read Archive (SRA) database, and SRA accession numbers for each sample is given in S1 Table.

The quality filtered and adapter trimmed reads were used as input in miRDeep [39], and each sample was analyzed individually to facilitate discovery of miRNAs expressed in particular tissues only (see methods). Initially miRDeep identified a total of 544 putative mature miRNAs (5p and 3p) and their corresponding precursor sequences in the Atlantic cod genome assembly with score values above our threshold settings (see methods). All the precursor sequences were subsequently analyzed by BLAST homology searches against miRBase. Any precursor sequence that showed a significant match (E-value ≤ 1E-06) to a miRNA precursor in miRBase was assumed to be the *Gadus morhua* ortholog of this miRNA precursor and subsequently annotated as the *Gadus morhua* ortholog of this miRNA family. The homology analysis revealed that most of the putative miRNAs discovered by miRDeep, a total of 219 Atlantic cod miRNA genes with 438 corresponding mature miRNAs (5p and 3p), could be classified as orthologs to evolutionary conserved miRNAs. The alignment of perfectly matching reads to the precursor of each miRNA gene (by miRDeep) also revealed whether there was 5p or 3p arm domination among the mature miRNAs processed from a given precursor. An overview of all precursor sequences along with their corresponding 5p and 3p mature sequences from all evolutionary conserved miRNA genes is given in S4 Table. Information about arm dominance of mature sequences and genome location of each miRNA gene is also given in this file.

A total of 50 precursors identified by miRDeep did not show significant matches in the homology analyses against miRBase. These were analyzed by additional BLAST homology searches against smallRNA databases as well as the Atlantic cod genome sequence. These BLAST searches revealed that there were three precursors with significant matches to other small RNAs in the Rfam database. These were likely to be other small RNAs rather than miRNA precursors and were removed. Several of the putative precursors also showed significant matches to multiple loci (>50) in the cod genome. These were likely to be interspersed repeats rather than miRNAs and, consequently, were removed from our group of putative novel miRNAs. The remaining precursor and corresponding mature sequences all showed characteristics expected from true miRNAs. These 26 miRNA precursor sequences with their 5p and 3p mature sequences are likely to represent novel miRNAs. An overview of precursor sequences from all such novel miRNA genes along with their corresponding 5p and 3p mature sequences, the observed arm dominance of mature sequences and genome location of each miRNA gene is given in S5 Table. The miRDeep analysis and subsequent validations, thus, discovered 245 miRNA genes in the Atlantic cod genome sequence with 490 5p and 3p mature miRNAs. After aligning miRNA gene family members that shared identical mature miRNAs (Sequencher software), there were 401 unique 5p or 3p mature miRNAs discovered in our materials.

**Fig 1:**
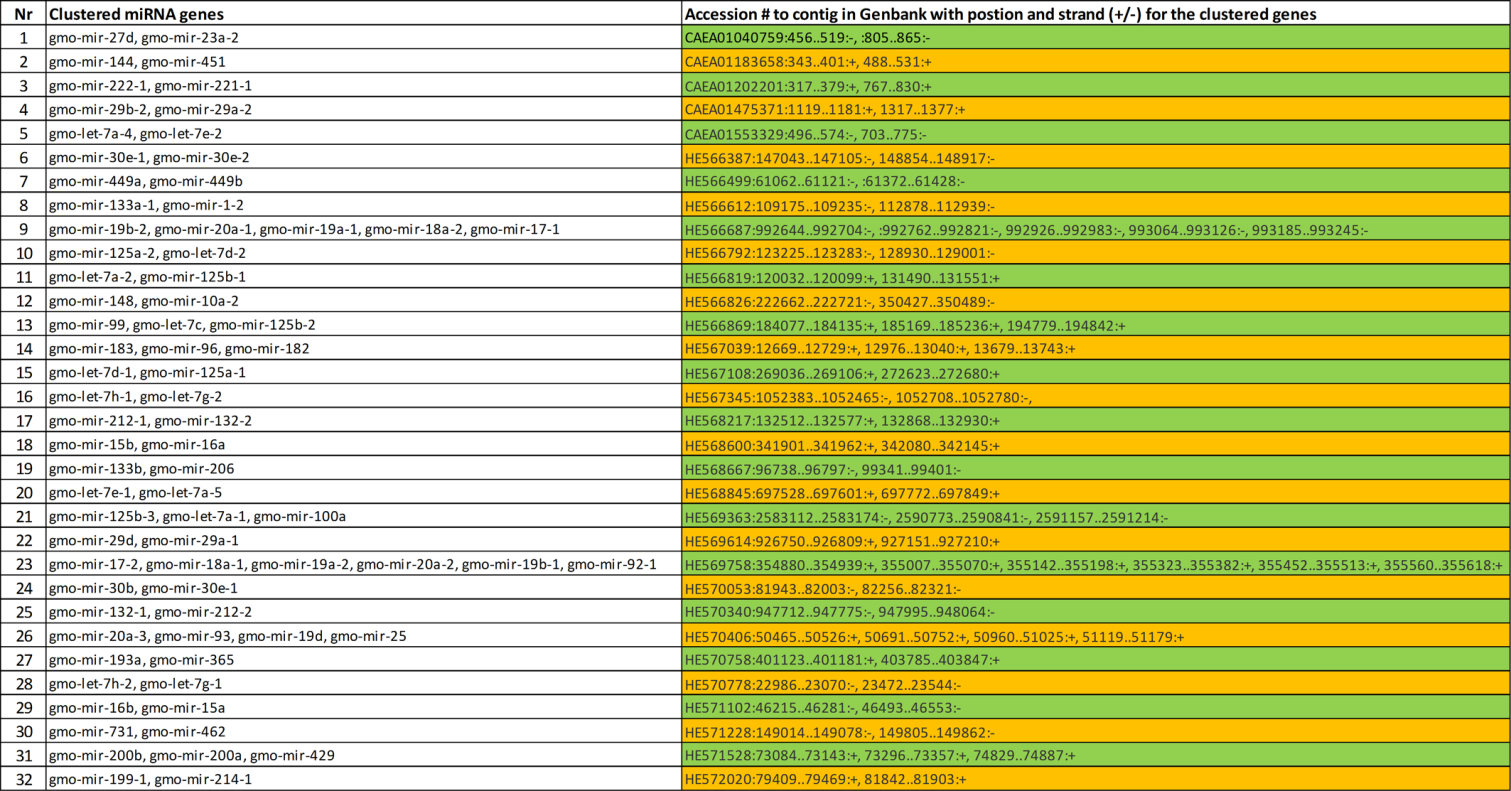
miRNA gene clusters in Atlantic cod.

A miRNA gene cluster is defined by miRBase as two or more miRNA genes located less than 10 kb apart and transcribed in the same direction. Comparing the location of the discovered miRNA genes within the current version of the Atlantic cod genome sequence showed that there were 32 such miRNA gene clusters, and a total of 77 of the 245 miRNA genes identified in our study were located in a gene cluster of from two to six miRNA genes. An overview of all clustered miRNA genes is given in Fig 1 and in S6 Table. All the miRNA genes in the gene clusters were evolutionarily conserved.

### Differentially expressed miRNAs validated by RT-qPCR

**Fig 2:**
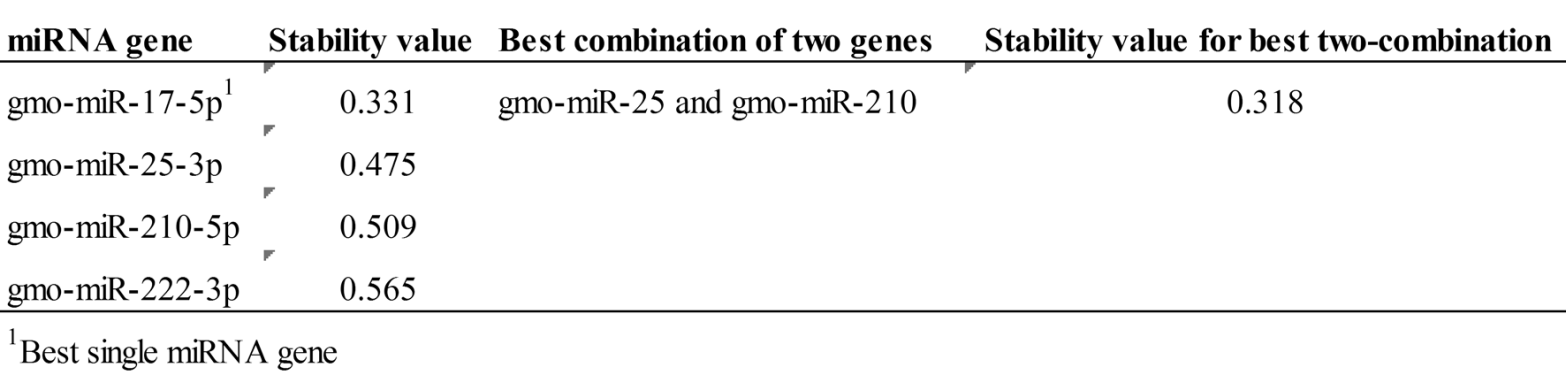
Estimated stability values by NormFinder.

**Fig 3:**
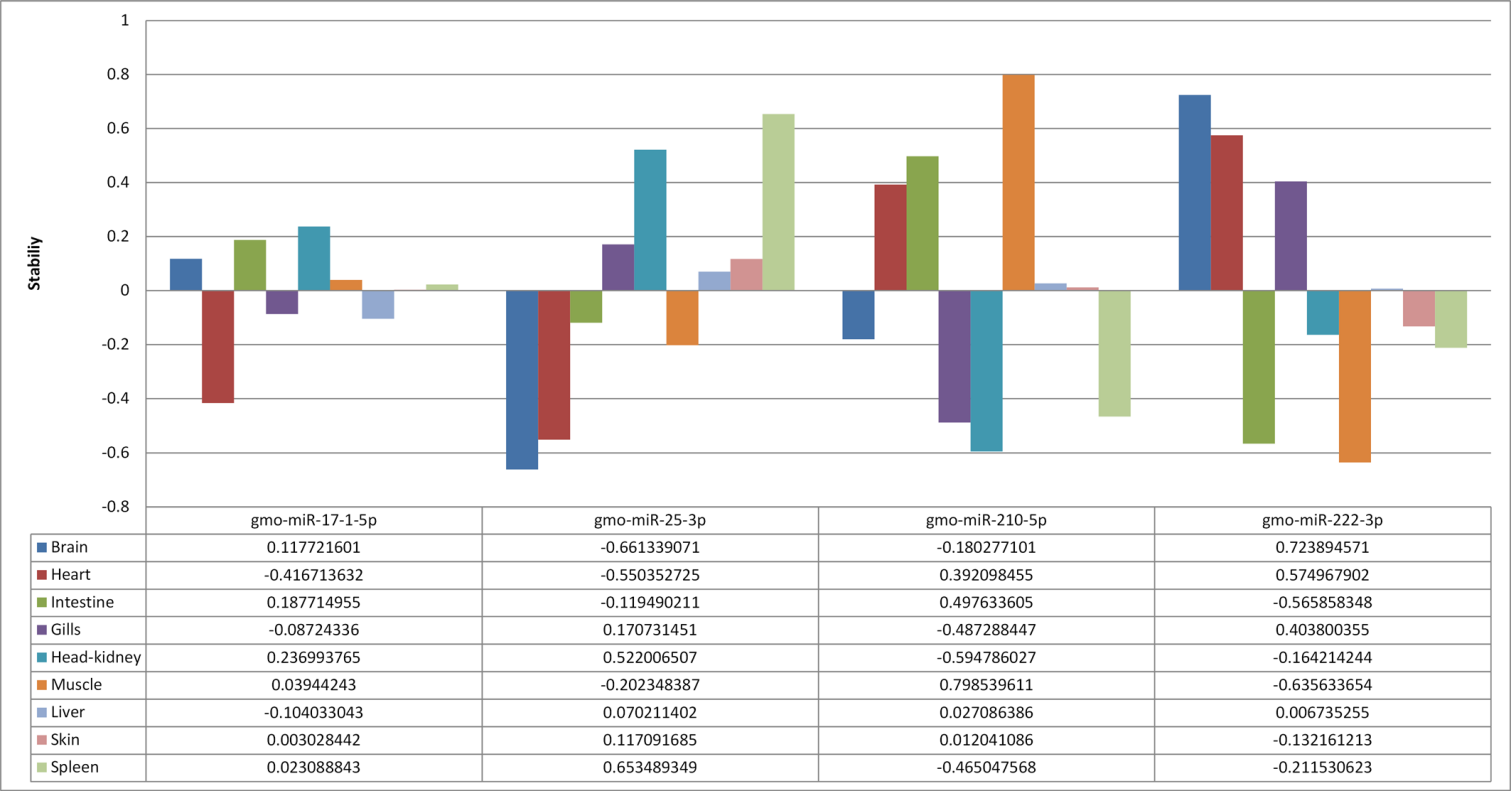
Variation across nine tissues for the four candidate reference genes. Fig3 shows the variation across tissues sampled from nine different organs for the four candidate reference genes.

Five candidate miRNAs were tested as endogenous controls (see methods for selection criteria). One of the selected assays for a candidate reference gene, *gmo*-miR-455-5p, showed suboptimal performance with primer-dimer products appearing at Ct >36 and was not further tested. The remaining four candidate reference genes (*gmo*-miR-17-1—5p, *gmo*-miR-25-3p, *gmo*-miR-210-5p and *gmo*-miR-222-3p) were successfully analyzed by RT-qPCR in all samples (n = 72). The results were used as input in NormFinder [37, 38] to estimate most stable single gene and most stable two-gene combination. The stability values for each single gene and most stable two-gene combination as calculated by NormFinder is given in Fig 2 while Fig 3 shows the variation in stability across all nine tissues for each candidate reference gene. The best performing single reference gene was *gmo*-miR-17-1—5p while the best two-gene combination, revealing a slightly better stability score, was *gmo*-miR-25-3p and *gmo*-miR-210-5p. Initial analysis of U6 showed an efficiency of 57% indicating that it was not suitable as endogenous control. RT-qPCR measurements of U6, *gmo*-miR-25-3p and *gmo*-miR-210-5p across seven samples showed a standard deviation (S.D.) of 2.2 for U6 while it was 1.4 for *gmo*-miR-25-3p and 1.1 for *gmo*-miR-210-5p. This indicated that, not only poor efficiency made U6 unsuitable as a reference gene, it did also show less stability (larger S.D.) in direct comparison with the best performing miRNAs. The two miRNAs, *gmo*-miR-25-3p and *gmo*-miR-210-5p were, therefore, applied as reference genes in our RT-qPCR method for measuring miRNA expression across, and within, tissues in Atlantic cod.

**Fig 4:**
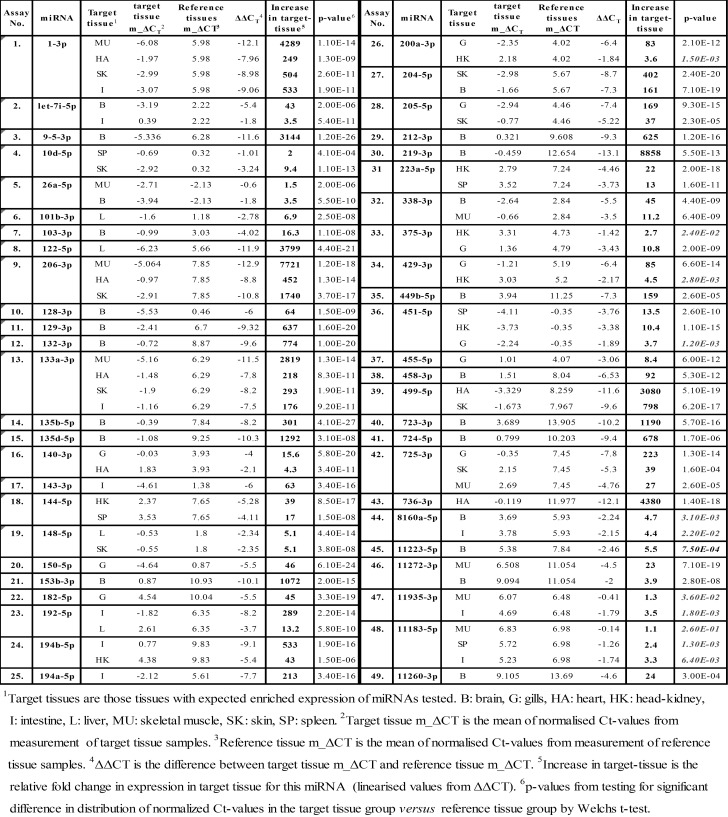
Summary of RT-qPCR results and tests to reveal differentially expressed miRNAs.

A total of 50 putatively differentially expressed miRNAs (see methods for selection criteria) were analyzed with our dedicated RT-qPCR method. Specific forward primers (S2 Table) were successfully applied in RT-qPCR assays for each mature miRNA except *gmo*-miR-7552. Forward primers failed to produce a single specific mature PCR-product in this assay, and *gmo*-miR-7552 was therefore not analyzed. Among the remaining miRNAs that were successfully analyzed by RT-qPCR, 45 out of 49 miRNAs showed a significantly higher expression level in one or more tissue. An overview of all miRNAs analyzed by qPCR, their target tissue(s), the relative increase in expression compared to reference tissues and p-values from t-tests is given in Fig 4. A correlation analysis between the relative increase of miRNAs from comparison of normalized reads and the relative increase of these miRNAs as measured by RT-qPCR showed a correlation coefficient of 0.63 S7 Table.

The magnitude of the increase in expression in target tissue(s) *versus* reference tissues were from rather small (1.5 fold) to very large differences with increases of several thousand-fold in some target tissues (e.g. *gmo*-miR-122 in liver). *Gmo*-miR-1 and *gmo*-miR-133a showed higher expression in four tissues (skeletal muscle, heart, skin and intestine). *Gmo-*miR-206 showed higher expression in tissues from skeletal muscle, heart and skin while *gmo*-miR-725 showed higher expression in skeletal muscle, skin and gills. Fifteen miRNAs showed higher expression in two out of the nine tissues tested, while the remaining 25 were highly expressed in tissue from one organ, only.

**Fig 5:**
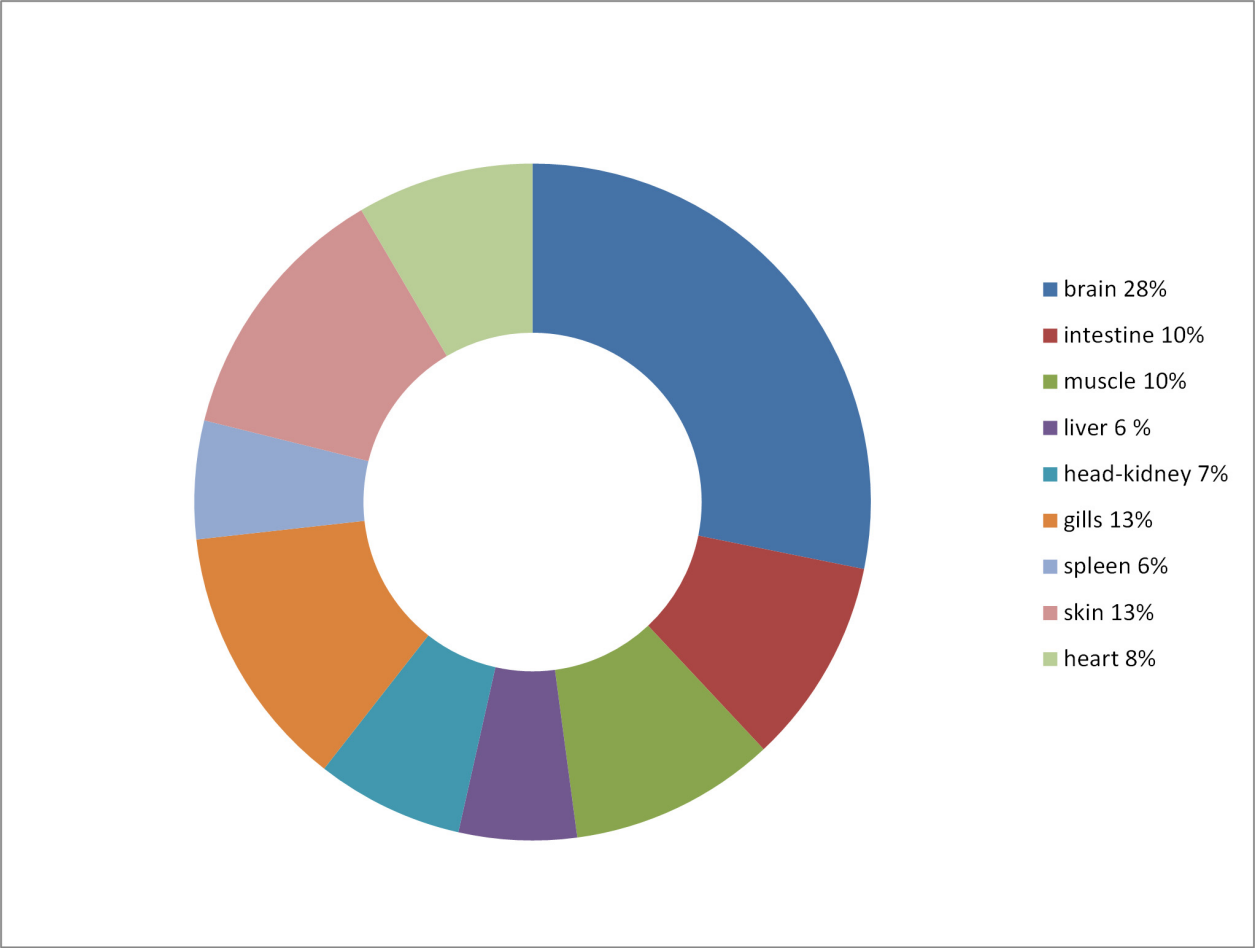
Distribution of differentially expressed miRNAs. The figure shows the distribution of differentially expressed miRNAs in nine different organ tissues from Atlantic cod.

Those differentially expressed miRNAs that were from closely located miRNA genes (gene clusters) showed, in general, similar high expression in the same tissues. *Gmo*-miR-1 and *gmo*-133a were e.g. highly expressed in the same four tissues (skeletal muscle, heart, skin and intestine). *Gmo*-miR-144 and *gmo*-miR-451 were highly expressed in head-kidney and spleen. *Gmo*-miR-212 and *gmo*-miR-132 were preferentially expressed in brain while *gmo*-miR-200a and *gmo*-miR-429 were highly expressed in gills. The proportion of miRNAs that were preferentially expressed in particular tissues is shown in Fig 5. This comparison showed that brain was the one tissue with largest proportion (28%) of differentially expressed miRNAs. The number of individuals in our study was small. Although we did not observe any differences in miRNA expression between subgroups of individuals (data not shown), we cannot rule out that the general miRNA expression, or expression of certain miRNAs may be affected by sex. The one individual that was deep sequenced showed Ct-values that grouped together with the remaining seven individuals indicating that a different sampling procedure did not affect the RT-qPCR measurements (data not shown).

**Fig 6:**
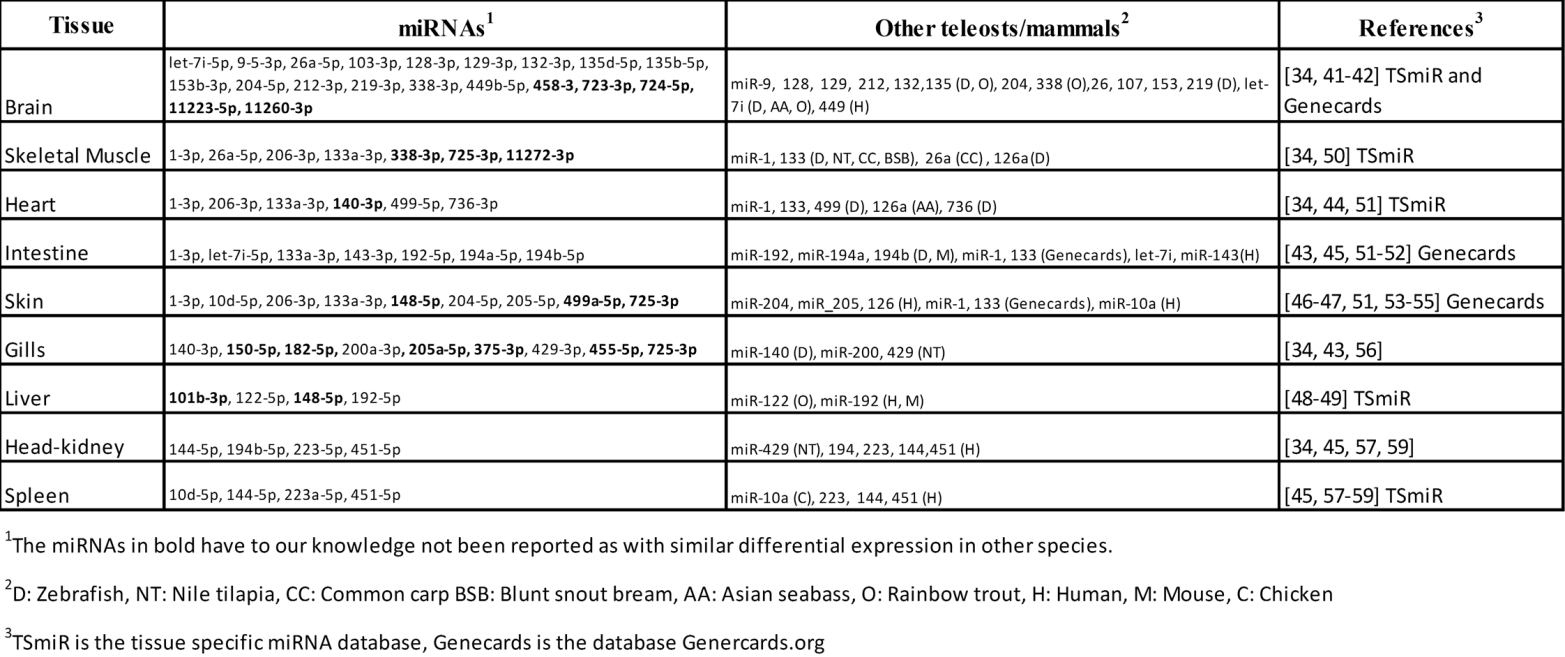
Differentially expressed miRNAs in Atlantic cod versus other teleosts or higher vertebrates.

A comparison to findings in other species could reveal whether the difference in expression of the particular miRNAs from our study are evolutionary conserved among vertebrates. Our results were, thus, compared to studies in other teleost as well as higher vertebrates (see references [34, 40–57]) and the miRNAs listed in databases TsmiR (http://bioeng.swjtu.edu.cn/TSmiR/) and Genecards (http://www.genecards.org/). A summary of this comparison showing the miRNAs preferentially expressed in each of the nine tissues and those miRNAs with similar finding in other species is given in Fig 6. This comparison revealed that most of the evolutionarily conserved miRNAs have been reported with similar expression patterns in other vertebrates.

Sixteen miRNAs (*Gmo*-miR-101b-3p, 140-3p, 148-5p, 150-5p, 182-5p, 205-5p, 338-3p, 375-3p, 455-5p, 458-3p, 723-3p, 724-5p, 725-3p, 11272-3p, 11223-5p, 11260-3p) have, to our knowledge, not been reported as with similar expression patterns in other vertebrates (all miRNAs shown in bold in Fig 6). Three of these (miR-723, 724 and 725) are teleost-specific, while three are novel miRNAs discovered in Atlantic cod (*gmo*-miR-11272-3p, 11223-5p and 11260-3p). Although their target transcripts are unknown, it is likely that they are regulators of genes/gene networks associated with the biological function of the tissue where they are preferentially expressed.

## Discussion

A recent study by Bizuayehu et al. [21] is, to our knowledge, the only miRNA discovery study in Atlantic cod. A total of 281 mature miRNA sequences (245 unique sequences) discovered in materials mainly from early developmental tissues were characterized in this study. We have characterized the sequences of 490 mature miRNAs (401 unique sequences). This represents a considerable increase in number of known mature miRNAs in Atlantic cod. A comparison to other fish species showed that all of the miRNA genes that were clustered in Atlantic cod were also clustered in a similar manner in other teleosts [14, 19]. The discovery that these evolutionarily conserved Atlantic cod miRNA genes are clustered as expected from other teleosts provides additional support that they are true miRNA genes in *Gadus morhua*. Comparing findings from both studies show that there is, presently, a total of 450 unique mature miRNAs (5p and 3p) characterized and annotated in Atlantic cod. Detailed and reliable knowledge of the mature miRNA sequences in a species is a prerequisite for studying miRNA expression by deep sequencing methods. The discovery and characterization of the miRNA repertoire in this study will therefore greatly benefit further functional studies of the miRNA transcriptome in Atlantic cod.

Quantitative studies of the miRNA transcriptome may be carried out by deep sequencing followed by confirmation of any miRNA expression differences by RT-qPCR. Measurements of miRNA expression by RT-qPCR require proper reference genes. The ideal reference genes are the ones with similar biological properties as the target genes except they are stably expressed in the species (or across materials) studied [37, 40]. The validation of reference genes in our study indicated that three miRNAs, either *gmo*-17-1—5p alone, or the combination of *gmo*-miR-25-3p and *gmo*-miR-210-5p, were particularly suitable as reference genes in Atlantic cod. In agreement with recent finding in similar studies [58] U6 performed less well compared to the miRNAs as an endogenous control. MiR-25-3p seems to perform well as a miRNA reference gene in several teleosts [37, 40].

Most of the miRNAs revealed as differentially expressed have been reported as to have similar expression patterns in other teleosts or higher vertebrates (Fig 6) [34, 40–57]. It is likely that these miRNAs, with both conserved mature miRNA sequences and enriched expression in same tissues across species, regulate the same gene pathways and, thus, have same functions in Atlantic cod as those revealed in other vertebrates. Among the miRNAs that may be of particular interest to the aquaculture industry are those regulating growth (miR-1 and miR-133) and lipid metabolism (miR-122) [34, 47–49]. Others of the differentially expressed miRNAs are known from other species to be important to health e.g. immune response to microbial infection in epithelial cells (let-7i) and scar damage repair (miR-10d) [44, 45, 51].

The differentially expressed miRNAs revealed in this study may be utilized to discover orthologous host genes in Atlantic cod. A host gene is a protein coding gene with one or more miRNA gene located within their introns. These host genes are usually expressed in a similar manner as the miRNA gene(s) that are located within their introns [13, 14]. Some miRNAs discovered in our study are located in well described host genes in other vertebrates. MiR-338 with preferential expression in brain in Atlantic cod is e.g. located in the gene brain apoptosis-associated tyrosine kinase (AATK) in other vertebrates [59]. Another miRNA showing significantly higher expression in skin (miRNA-204) is known from other species to be located in the host gene TRPM3 [13]. As the location of the Atlantic cod miRNA genes in our study are known, this may facilitate characterization and annotation of such orthologous host genes in the Atlantic cod genome.

## Conclusions

The number of known mature miRNAs was considerably increased by our discovery and characterization of miRNAs and miRNA genes in Atlantic cod. This will benefit further functional studies of miRNA expression using deep sequencing methods. The validation study identified suitable endogenous controls and showed that stable miRNAs are suitable reference genes for RT-qPCR analysis of miRNA expression. Applying the RT-qPCR method we identified several miRNAs likely to have important regulatory functions in particular organs.

### Ethical considerations

Dissection of fish and sampling of materials was performed in agreement with the provisions enforced by the Norwegian Animal Research Authority.

## Supporting Information

S1 Table The table shows sequence Read Archive (SRA) database accession numbers to all samples sequenced using the Illumina small RNA sequencing platform. (XLSX)

S2 Table The table shows all forward primer sequences used in the miRNA-qPCR assays. (XLSX)

S3 Table The table shows read counts of mature miRNAs from analysis with Novoalign. (XLSX)

S4 Table The table shows all evolutionary conserved miRNAs identified and characterized in Atlantic cod. (XLSX)

S5 Table The table shows all novel miRNAs identified and characterized in Atlantic cod. (XLSX)

S6 Table The table shows all genes located in gene clusters. (XLSX)

S7 Table Correlation between relative increase of differentially expressed miRNAs in RT-qPCR measurements vs estimates of the relative increase in deep sequencing samples. (XLSX)
